# Hemp in Veterinary Medicine: From Feed to Drug

**DOI:** 10.3389/fvets.2020.00387

**Published:** 2020-07-28

**Authors:** Giorgia della Rocca, Alessandra Di Salvo

**Affiliations:** Dipartimento di Medicina Veterinaria, Centro di Ricerca sul Dolore Animale (CeRiDA), Università degli Studi di Perugia, Perugia, Italy

**Keywords:** cannabis, cannabidiol (CBD), pharmaceutical activity, nutraceutical activity, nutritional properties, tetrahydrocannabinol (THC), veterinary medicine

## Abstract

Hemp (*Cannabis sativa*) is an angiosperm plant belonging to the *Cannabaceae* family. Its cultivation dates back to centuries. It has always been cultivated due to the possibility of exploiting almost all the parts of the plant: paper, fabrics, ropes, bio-compounds with excellent insulating capacity, fuel, biodegradable plastic, antibacterial detergents, and food products, such as flour, oils, seeds, herbal teas, and beer, are indeed obtained from hemp. Hemp flowers have also always been used for their curative effects, as well as for recreational purposes due to their psychotropic effects. Cannabis contains almost 500 chemical compounds, such as phytocannabinoids, terpenes, flavonoids, amino acids, fatty acids, vitamins, and macro-, and micro-elements, among others. When utilized as a food source, hemp shows excellent nutritional and health-promoting (nutraceutical) properties, mainly due to the high content in polyunsaturated fatty acids (especially those belonging to the ω-3 series), as well as in phenolic compounds, which seem effective in the prevention of common diseases such as gastrointestinal disorders, neurodegenerative diseases, cancer, and others. Moreover, hemp oil and other oils (i.e., olive oil and medium-chain triglyceride–MCT–oil) enriched in CBD, as well as extracts from hemp dried flowers (*Cannabis* extracts), are authorized in some countries for therapeutic purposes as a second-choice approach (when conventional therapies have failed) for a certain number of clinical conditions such as pain and inflammation, epilepsy, anxiety disorders, nausea, emesis, and anorexia, among others. The present review will synthetize the beneficial properties of hemp and hemp derivatives in animal nutrition and therapeutics.

## Introduction

Hemp, or *Cannabis*, is an annual cycle angiosperm plant, belonging to the *Cannabaceae* family (1). The terms “hemp” and “*cannabis*” are synonymous, but usually hemp refers to the plant as a vegetable, textile fiber, or raw material for construction or in the gastronomic field; the term cannabis is preferred instead when it is intended to emphasize its therapeutic or psychoactive connotation. From a botanical point of view, however, the plant is the same: *Cannabis sativa* (sativa = “useful”), with all its subspecies and varieties (2).

At present, there are two main hemp cultures. The first is industrial hemp (also called “*Cannabis light*”), i.e., the one with a THC level lesser than 0.2%). Industrial hemp has countless uses: specific parts of the plant (e.g., external fibers of the stem, seeds) are in fact used: in the textile and paper industry, to produce fabrics, cloths, ropes, and paper; in construction, as a bio-compound with excellent insulating capacity that can be used as a filler for walls, insulation for roofs, for external and internal walls, and for floors; to produce biodegradable plastic and antibacterial detergents; and in feed, as flours, oils, seeds, herbal teas, beer, etc. The second culture is the so-called medical cannabis, which meets the quality standards for the use of the products as a medicine; in this case, the dried plant's inflorescences and apical leaves are used. In medicine, Cannabis is used for its supposed therapeutic properties, which guarantee its effectiveness in the treatment of a wide range of pathologies, both in humans in and animals.

Hemp contains in its various components a considerable number of active ingredients mainly represented by phytocannabinoids, terpenes and terpenoids, and flavonoids (the main ones are listed in Table 1), as well as saturated and unsaturated fatty acids, proteins, carbohydrates, chlorophylls, vitamins, and minerals, among others (3).

**Table 1:** Main phytocannabinoids, terpenes, and flavonoids contained in *Cannabis Sativa* spp. (3, 4).

**Phytocannabinoids**	**Terpenes**	**Flavonoids**
Delta-8-tetrahydrocannabinol (Δ^8^-THC)	Limonene	Cannaflavin
Delta-9-tetrahydrocannabinol (Δ^9^-THC)	Myrcene	Quercetin
Delta-9-tetrahydrocannabivarin (Δ^9^-THCV)	α-Pinene	Luteolin
Cannabidiol (CBD)	Caryophyllene	Kaempferol
Cannabinol (CBN)	Linalool	
Cannabigerol (CBG)		
Cannabichromene (CBC)		
Cannabinodiol (CBND)		
Cannabicyclol (CBL)		
Cannabielsoin (CBE)		
Cannabitriol (CBT)		

Many of the constituents of *C. sativa* can be classified as either nutrients, nutraceuticals, or pharmaceutical ingredients (5).

The present review synthetizes the main nutritional, nutraceutical, and pharmaceutical properties of *C. Sativa* spp.

## Nutritional Properties of Cannabis

*C. sativa* has been an important source of food in the Old World, as hempseeds and seed meal are excellent sources of dietary oil, fiber, and protein. After the prohibition of the cultivation of all Cannabis varieties in the late 1930s, the potential use and development of hempseed as a food for humans and domesticated animals was interrupted. During the last two decades, thanks to law changes, hemp with a THC content lower than 0.2% has been reconsidered as a valuable industrial crop for food in some countries, and hempseed and hempseed food products have become available as food for both humans and domesticated animals (6).

Although the nutritive content of hemp may change by virtue of different hemp cultivars, both hempseed and hempseed meals are rich sources of protein, polyunsaturated oils, vitamins, and useful minerals (6).

Whole hempseed generally contains about 20–25% proteins, 25–35% carbohydrates (with 10–15% insoluble fiber), 25–35% oil (obtained from cold pressing of seeds or by extraction), vitamins, and minerals (6–8). The two main proteins in hempseeds are edestin and albumin, both containing significant amounts of all essential amino acids. In addition, hempseeds contain the amino acids arginine and glutamic acid at exceptionally high levels, as well as good amounts of the sulfur-containing amino acids methionine and cystine (6).

The oil obtained from hempseed contains up to 90% of polyunsaturated fatty acids (PUFA) and is characterized by a great amount of the two essential fatty acids (EFAs) linoleic acid (LA, 18:2 ω-6; average amount 56%) and α-linoleic acid (ALA, 18:3 ω-3; average amount 16%), resulting in a 3.5:1 ratio of ω-6 to ω-3. This ratio is considered very beneficial for the humans' diet (8). Hempseed oil also tends to contain high amounts of metabolites of LA and ALA, such as γ-linolenic acid (GLA, 18:3 ω-6) and stearidonic acid (SDA, 18:4 ω-3), respectively (6). Although these metabolites are not considered EFAs, their supplementation in the diet can be extremely beneficial.

Following the removal of the lipid fraction from seeds, hemp cakes or meal can be obtained. Hemp meal has a major protein content (up to 40%) and obviously a reduced amount of lipids (10–11%) (6, 9). Hempseeds and hempseed cakes contain tocopherols, especially γ-tocopherol (60.85 and 33.72 mg/100 g dry matter, respectively) (10).

According to the scientific opinion of the European Food Safety Authority (EFSA) panel on Additives and Products or Substances used in Animal Feed, hempseed and hempseed cakes could be used as feed materials for all animal species, although with specie-specific differences with regard to the rate of inclusion in the diet (11). Hemp oil can be used as a supplement in feed mixtures for animals as a rich source of essential fatty acids, while seeds and hempseed cakes can be used as a fat and protein source in animals' diets.

Several studies have evaluated the effects of the intake of hempseed or its products in farm animals, albeit with results not always conclusive. Here is a synthesis of the most indicative studies, divided by animal species.

### Poultry

#### Broilers

Following the incorporation of *C. sativa* seeds at rate of 10 and 20% to a basal feed, a significant increase in body weight was observed in broilers compared to animals fed with the basal feed only. The feed intake was lower, and the feed conversion rate better in animals with the diet containing hempseed than in the control group. The best growth performance was achieved with the higher amounts of hempseeds. Conversely, at concentrations of hempseeds lower than 5%, the broilers' body weight was significantly lower than in control group (12).

A reduction in average daily feed intake and average daily gain was also observed by Mahmoudi et al. (13) in broilers fed with 2.5% of hempseeds during the first 21 days of treatment, while no difference resulted in body gain with diets at 4 and 7.5% (13, 14).

In the studies by Eriksson and Wall (15) and Jing et al. (16), the incorporation of neither hemp oil up to 6% nor hempseed cakes at 10 and 20% ameliorated the growth performance of chickens.

Štastník et al. (17) studied the addition of 5 and 15% of hempseed cakes in diets for broilers. With the higher dose, the authors observed a negative effect on broilers' growth, with no differences in carcass weight and percentage of breast meat and thigh meat, compared to diets without hempseed cakes.

#### Laying Hens

In the study by Silversides and Lefrançois (18), hens' performance was evaluated following inclusion in the diet of hempseed meal at concentrations of 5, 10, and 20%. No differences were observed in body weight, feed consumption, feed efficiency, and egg production compared to a control group.

Similar results were reported for diet containing hempseeds from 3 to 30%, hempseed cakes at 20%, and hemp oil up to 12% (16, 19–22), with the exception of a lower feed intake in hens fed with hemp oil at 4% (20) and a major egg production after a diet containing 3% of hempseeds (22).

Gakhar et al. (19) observed a greater egg mass after a diet with 20% hempseeds compared to that obtained with a minor content or without hempseeds; no difference was observed in feed intake and body weight or egg quality such as eggshell thickness, albumen height, or specific gravity.

Significantly greater egg mass was also observed after a diet with hempseeds at 8%, and a greater egg weight was obtained with a feed containing from 3 to 9% of hempseeds compared to a control diet (21, 22).

Conversely, during the first weeks of a study conducted by Neijat et al. (20), a lower weight of eggs laid from hens fed with hempseed 30% was observed compared to eggs laid in the control group and in groups fed with lower concentrations of hempseed; the authors concluded that this difference was due to the adaptation to the diet because at the end of the study no differences resulted among groups.

Overall, the majority of authors concluded that the incorporation of the hemp product to the diet of poultry has no negative effects on their performances.

Several studies have also investigated the effects of hemp addition on egg content of EFAs, PUFA, and saturated and monounsaturated fatty acids (SFA, MUFA).

In the study by Halle and Schöne (23), the addition to the diet of 5, 10, or 15% hempseed cakes resulted in a linear increase in the concentrations of LA and ALA, with decrease in SFA and MUFA.

Neijat et al. (24) evaluated the addition of either hempseeds (10, 20, and 30%) and hempseed oil (4.5 and 9.0%) and found a significant increase in egg yolks of ALA and docosahexaenoic acid (DHA) compared to a control group for the highest levels of hempseeds and hempseed oil.

In the study by Mierlit ´ ǎ (21), egg yolk fatty acid profile differed when hemp oil or hempseed cakes were added to the diet: LA was increased with hempseed cakes compared to hempseeds and the control group, while ALA was higher compared to control and lower compared to the hempseed group. Concentrations of oleic acid were lower in yolk when hens were fed with both hemp derivatives, leading to lower MUFA concentrations.

In the same study, Mierlit ´ ǎ (21) found higher concentrations of α-tocopherol, indicating for a better antioxidant capacity, in eggs from laying hens fed with a diet added with hempseeds or hempseed cakes.

Finally, Raza et al. (25) found that the addition of 25% hempseed in the diet of hens improved the ω-6/ω-3 ratio in the yolk of eggs.

### Ruminants

#### Dairy Cow and Cattle

Karlsson et al. evaluated the effect of different amounts (143, 233, and 318 g/kg dry matter) of hempseed cakes in the diet of dairy cows. Milk yield was higher when the cows were fed with addition of 143 g/kg compared to controls and cows fed with higher levels of hempseed cakes. Similarly, the efficiency of converting dietary crude protein into milk protein decreased with every additional level of hempseed cakes, leading the authors to conclude that inclusion of 233 or 318 g/kg had no benefits in milk performance (26).

In other studies, the addition of whole hempseeds or hempseed cakes in cattle's diet did not show differences in weight gain when compared with animals fed with “standard diets” (27, 28). However, as reported by Mustafa et al. (29), hempseed meal can be considered as an excellent natural source of rumen undegraded crude protein. In calves and steers fed with hempseed cakes as a protein source, a lower number of long particles in feces and, only for steers, a higher consistency of feces were observed compared to animals fed with a mixture of soybean meal and rolled barley. Putting all together, these observations are indicative for a better rumen functionality probably due to a higher fiber content and/or a minor starch amount in hempseed cakes compared to control diets (28).

#### Ewes

Two studies have been conducted by Mierlit ´ ǎ (10, 30) in the mid-lactating ewes in order to evaluate the effect of dietary supplementation with hempseeds or hempseed cakes. In the first study (30), the addition to the diet with 25% hempseeds increased the fat content in milk, without affecting the milk protein content. In the second study (10), both hempseeds (180 g/d) and hempseed cakes (480 g/d) added to feed determined an increase in milk fat and milk yield when compared to controls. Both hempseeds and cakes increased the concentration of ALA in ewes' milk by 66 and 49%, respectively, together with an increase in PUFA, MUFA, and long-chain fatty acids and a decrease in total SFA, short-chain fatty acids, and medium-chain fatty acids. Moreover, the addition of hempseeds or hempseed cakes to the sheep's diet preserved the oxidative stability of milk, as determined by a higher α-tocopherol concentration in sheep milk and higher total antioxidant capacity (evidenced by lower malondialdehyde concentration in sheep milk).

#### Goats

Cozma et al. studied the effects of the addition of hempseed oil in a hay-based diet for dairy goats. Hempseed oil was added in the concentrate mixture by 4.70% during 31 days of the experiment. This addition did not influence milk yield, while milk fat content was higher compared to the control group, with increasing conjugated fatty acid and PUFA proportions (31).

### Pigs

Mourot and Guillevic (32) evaluated the effects on growth performance, body composition, and meat quality parameters of three diets containing either hempseed oil, palm oil, or rapeseed oil and found no differences with regard to the added oil.

## Nutraceutical Properties of Cannabis

Some studies have also evaluated the nutraceutical effects of the industrial hempseed use in animal feeding.

A beneficial effect of the addition of hempseeds in the diet of broilers and hens resulted in an increase in the breaking strength tibia and in a reduction in the rate of its deformation compared to animals fed with a hemp-free diet (14, 22). This feature is very important, as bone fractures in poultry farm have a huge impact on the mortality rate and are a concern for animals' welfare (33).

Another beneficial effect of the use of hempseeds in the alimentation of broilers is an ameliorated serum lipidic profile with an increase in high-density lipoprotein (HDL) values and a reduction in triglycerides, low-density lipoproteins (LDL), and total cholesterol amounts (13). A better serum lipidic profile was also observed in hens (34).

Some authors speculated that hempseeds and hemp oil could have a protective effect on the development of liver diseases in hens and chickens by virtue of a reduction of hepatic enzymes observed following their inclusion in the diet (20, 34). Differently, the inclusion of hempseed oil in the diet of goats produced a significant increase in serum total lipids compared to animals fed with a control feed; however, alanine aminotransferase and γ-glutamyltransferase values did not differ from that observed in the control group, indicating the probable absence of the deleterious effect of hemp oil on liver health (31).

The antimicrobial activity of cannabis derivatives has also been investigated. Essential oils derived from Cannabis has shown *in vitro* antimicrobial properties on several bacteria (34, 35), with an observed favorable effect on microbial colonization of poultry gut following the addition of hempseeds (alone or in combination with dill seeds) to the basal feed: the count of the pathogen *E. coli* was reduced and that of *Lactobacillus* increased compared to the control group. However, in another study, the administration of hempseed expellers to chickens did not reveal differences in the counts of microorganisms such as *E. coli, Lactobacillus*, and *Enterococci* spp. compared to a basal diet (36). Similarly, no significant difference in the count of *C. perfringens* in caeca of broilers was observed by Eriksson and Wall (15) comparing diets with and without hempseed cakes.

Recently, Iannaccone et al. (37) analyzed the gene expression of whole blood from lactating ewes fed with a basal diet or a supplementation of hempseed at 5%, finding an upregulation of genes associated with energy production and to thermogenesis activation (that could make animals more easily adaptable to cold temperature) in the last group.

Palade et al. (38) have hypothesized that the inclusion of hempseed meal in the diet of sows during the later period of gestation and in early weeks of lactation promotes antioxidative systemic status and that this effect could be transferred to sucking piglets by milk.

Finally, an anti-inflammatory activity of Cannabis derivatives has also been hypothesized (5), due to the proper ratio (3:1) of ω-6 to ω-3 fatty acids of Cannabis seed oil. As the pro-inflammatory eicosanoid cascade is promoted by a high ω-6/ω-3 fatty acid ratio (20:1) that results in increased prostaglandin 2α (PG2α), diets rich in ω-3 and poor in ω-6 may have a less pro-inflammatory profile (39).

## Pharmaceutical Properties of Cannabis

In recent years, interest in the possible pharmacological use of compounds based on extracts of *C. sativa* has been increasing. Among the several phytocannabinoids contained in Cannabis, the main (but not unique) responsible for their therapeutic effects are Δ9-tetrahydrocannabinol (THC), which is also responsible for the psychotropic effect of the plant, and cannabidiol (CBD), which is not psychoactive and seems able to mitigate the psychotropic activity of THC (40). According to some authors, the presence of other phytocannabinoids as well as terpenes and flavonoids also contributes to the therapeutic activity of the two mentioned phytocannabinoids. In other words, the phytocomplex would appear pharmacologically more active than the individual components, thanks to the so-called *entourage effect*, which translates into an increasing affinity of endogenous endocannabinoid mediators for their receptors and a slowdown in their metabolic inactivation (4, 41). However, the currently available scientific studies do not fully support this hypothesis yet (42).

The biological activities of phytocannabinoids are expressed, thanks to their ability to interact with the endocannabinoid system (ECS), represented by the ensemble of cannabinoid receptors, endocannabinoids (compounds produced by the body that bind to the aforementioned receptors), and enzymes responsible for their synthesis and their catabolism and genes coding for these proteins.

The presence of the ECS has been identified in numerous animal species, such as invertebrates (43) including mollusks (44), protozoa (45, 46), nematodes, onychophorans, crustaceans (47), sea urchins (48), and some species of cnidarians (47, 49); lower vertebrates including amphibia (salamanders, frogs) and other aquatic organisms (43) [such as goldfish (50) and zebrafish (51)]; and vertebrates like poultry (43) [such as pigeons (52), chickens, zebra finch]; and mammals (52). With specific regard to companion animals, in dogs the presence of cannabinoid receptors or their ligands has been identified in skin and skin appendages (53–55), gastrointestinal tract (56, 57), central and peripheral nervous system (58–60), joints (61), and embryo (62); in cats, cannabinoid receptors have been detected in the brain (63), skin (64), and ovary and oviduct (65).

Due to the presence of ECS in many body tissues, it has been possible to hypothesize first and then confirm (thanks to numerous studies conducted in animal models) its role in many physiological functions. In fact, this system seems capable of modulating the synaptic responses and, depending on the population of involved cells, influencing numerous biological functions, sometimes even in a variable way. In particular, ECS seems to be involved in functions that underlie memory, learning, and coordination of motor functions, as well as to perform pain-relieving, anti-inflammatory, antioxidant, immunosuppressive, hypotensive, antiemetic activity, and regulation of sleep, appetite, and reproductive functions. Finally, recent studies are investigating a possible involvement of ECS in neuroprotection and in the control of the proliferation of cancer cells (66, 67).

Given the role of the ECS in all these biological functions, it seems strongly likely that a plant that contains active ingredients capable of interacting with it (thanks to both a direct action on the receptors and following the “*entourage effect*”) can play as many modulation roles, which can be exploited pharmacologically in a multiplicity of pathologies not always responsive to conventional treatments.

In humans, the use of cannabis is proposed in conditions such as pain associated with spasticity in multiple sclerosis, neuropathic pain, anorexia, nausea, and vomiting associated with certain pathologies or their treatment, some forms of glaucoma, and epilepsy. Other possible therapeutic uses may concern anxiety and insomnia, functional or autoimmune intestinal disorders, musculoskeletal problems such as fibromyalgia, and, finally, some types of cancer, among others (68).

In veterinary medicine, albeit empirically, many veterinarians successfully use cannabis derivatives for pain control (mainly for neuropathic, osteoarthritis, and cancer pain), idiopathic epilepsy refractory to conventional treatments, allergic skin diseases, and mood disorders (e.g., anxiety) and for a series of other pathological conditions affecting dogs and cats (5, 69). A detailed overview on the endocannabinoid system; the biology, chemistry, and history of cannabis; and how animal species interact with various formulations and cannabis treatments is proposed by Hartsel et al. (5), Landa et al. (70), and Silver (71). The use of cannabis derivatives seems not associated with the occurrence of side effects, even when carried out for long periods (72–74).

However, to date relatively few studies have investigated the efficacy, tolerability, and pharmacokinetics of cannabis derivatives in animals of veterinary interest.

### Efficacy Studies

At present, only two published studies have evaluated the efficacy of Cannabis derivatives for the treatment of osteoarthritis (OA) and epilepsy, respectively.

Gamble et al. (75) performed a randomized, double-blind, crossover, placebo-controlled trial that involved 16 dogs with radiographic evidence of OA, owner-reported pain signs, lameness, and pain on joint palpation. The product under study was an industrial hemp extract reconstituted in olive oil, containing mainly CBD together with low concentrations of other phytocannabinoids. Dogs received 2 mg/kg of CBD or placebo orally every 12 hours for 4 weeks. Before the beginning of the treatment (baseline) and at 2 and 4 weeks of therapy, the degree of pain, the level of activity, the degree of lameness, and limb bearing were evaluated. Dogs treated with CBD showed a significant reduction in pain and increase in activity compared to both baseline and placebo, although no significant differences were observed regarding the degree of lameness and the limb bearing.

To assess the effect of CBD administration in addition to conventional antiepileptic treatment on seizure frequency in dogs, McGrath et al. (74) evaluated 26 client-owned dogs with intractable idiopathic epilepsy in a randomized blinded controlled clinical trial. Two groups of subjects were treated orally with 2.5 mg/kg of CBD every 12 h or with placebo for 12 weeks, respectively. Dogs in the CBD group showed a significant reduction in seizure frequency (median change = 33%) compared to the placebo group. However, the proportion of dogs considered treatment responders (≥50% reduction in seizure activity) was similar between groups. The authors therefore concluded that further research is needed to determine whether a higher dose of CBD would be effective in reducing seizure activity by ≥50%.

### Pharmacokinetic Studies

At present, pharmacokinetic studies in animals are still limited and referring only to the canine (72, 75–80) and feline (72) species. The results of these studies are summarized in Table 2.

**Table 2:** Summary of studies evaluating the pharmacokinetics of *Cannabis* derivatives in dogs and cats.

**Number of dogs/cats**	**Drug/formulation**	**Dose/route and frequency of administration**	**Main findings**	**References**
6 dogs (16–24 kg)	CBD gelatin capsules	180 mg *per os* Single administration	CBD was not detected in three dogs over the 24-h collection period. In the other three dogs, CBD plasma levels were low and could only be determined over limited periods of time. These low plasma concentrations produced individual bioavailability values of 13, 13, and 19%, respectively	(76)
2 dogs (47 and 27 kg, respectively)	Bediol® oil (6,8% THC, 8% CBD)	Dog #1: THC 9 mg; CBD 11,5 mg *per os* Dog #2: THC 3,6 mg; CBD 4,6 mg *per os* Single administration	Peak concentrations for both THC (6.0 and 3.3 ng/mL) and CBD (9.3 and 4.6 ng/mL) were observed 1 h after Bediol administration and followed by a slow elimination phase along with the 12 h collection period; at 12 h post-administrations, both dog #1 and #2 came back to THC and CBD values comparable to those prior Bediol administration	(77)
30 dogs (10/group) (9.5–16.2 kg)	Microencapsulated CBD oil beads CBD-infused oil CBD-infused transdermal cream	75 and 150 mg *per os* or transcutaneously Single and multiple (twice a day for 6 weeks) administration	After the first administration, the mean Cmax of CBD was higher, with both doses administered and at all withdrawal times, in dogs that received the infused-oil formulation (625.3 and 845.5 ng/ml for 75 and 150 mg, respectively) and lower in those treated with transdermal cream (74.3 and 277.6 ng/ml). With beads, the Cmax stood at intermediate values (346.3 and 578 ng/ml). Following prolonged treatment, similar concentrations (slightly higher or lower) with infused oil (649.43 and 903.68 ng/ml) and beads (364.93 and 546.06 ng/ml) were detected, while with the transdermal cream, much lower values were obtained compared to those detected after a single treatment (30.10 and 97.46 mg/ml). In addition to guaranteeing CBD plasma concentrations higher than the other two routes with both dosages used, the infused-oil formulation presented the least interindividual variability. With infused oil, the T1/2 el after administration of 75 and 150 mg was equal to 199.7 and 127.5 min, respectively, against values of 95.4 and 115.9 min obtained with beads	(78)
8 dogs (4/group) (10.7–11.9 kg)	CBD oil	2 and 8 mg/kg *per os* Single administration	The median CBD Cmax obtained with the two dosages used (2 and 8 mg/kg) was 102.3 ng/ml (range: 60.7–132.0 ng/ml) and 590.8 ng/ml (range: 389.5–904.5 ng/ml) and was reached after 1.5 and 2 h, respectively. The T1/2 el of CBD was 4.2 h for both dosages (range: 3.8–6.8 and 3.8–4.8 h for 2 and 8 mg/kg, respectively)	(75)
6 dogs (~30 kg) Fed and fasting	Bedrocan® oil (20% THC, 0.5% CBD)	THC 1.5 mg/kg; CBD 0.037 mg/kg *per os* Single administration	CBD was below the detection limit of the analytical method used (1 ng/ml). THC was quantifiable from 0.5 to 10 h after administration, although there was a large inter-subjective variability. Fasting dogs showed a shorter absorption phase (Tmax: 1.25 vs. 5 h) and a higher maximum plasma concentration (Cmax: 24 vs. 7.1 ng/ml) compared to the fed group. A higher AUC was obtained in fasting dogs compared to the fed ones (75.25 vs. 29.74 ng/mL*h, in which the relative oral bioavailability was 48.22%	(79)
6 dogs (7.5–12 kg)	CBD oil	2 mg/kg *per os* Single administration	Pharmacokinetics revealed a mean Cmax of 301 ng/mL, an AUC of 1297 ng/mL*h, a Tmax of 1.4 h, and a T1/2 el of 1.0 h	(72)
6 dogs (11–13 kg)	Sativex®	3 sprays (8.1 mg of THC and 7.5 mg of CBD) Sublingual Single and multiple (3 sprays daily for 14 days) administration	Cmax of both THC and CBD were achieved 2 h (Tmax) after administration in the single-dose treatment (THC: Cmax = 18.5 ng/mL; CBD: Cmax = 10.5 ng/mL) and 1 h (Tmax) after the multiple dose treatment (THC: Cmax = 24.5 ng/mL; CBD: Cmax = 15.2 ng/mL). A potential progressive accumulation of both CBD and THC was detected following repeated exposure, with maximum plasma concentrations for both cannabinoids being achieved following multiple doses. AUC values in the multiple-dose condition were higher compared to the single-dose treatment, being 165.0 ng/mL*h for THC and 123.1 ng/mL*h for CBD.	(80)
6 cats ((3.3–5.2 kg)	CBD oil	2 mg/kg *per os* Twice daily for 12 weeks	Pharmacokinetics revealed a mean Cmax of 43 ng/mL, an AUC of 164 ng/mL*h, a Tmax of 2 h, and a T1/2 el of 1.5 h.	(72)

*AUC, area under curve; Cmax, maximum (serum) concentration; Tmax, time to maximal (serum) concentration; T1/2 el, half-life of elimination*.

From a summary of the pharmacokinetic data obtained by the aforementioned authors, some practical considerations arise:

- Although the blood concentrations obtained are rather low being in the order of ng/ml, it is likely that these concentrations are still effective, since neurotransmitters usually act at infinitesimal concentrations.
- The low bioavailability obtained after oral administration is presumably due to a first-pass effect (liver metabolism before the drug enters the systemic circulation) and the type of formulation used.
- The oil formulations guarantee a better pharmacokinetic profile (both in terms of bioavailability and half-life).
- To promote better oral absorption of the active ingredients, cannabinoids should be administered in fasted animals.
- The elimination kinetics of cannabinoids is such as to suggest administrations every 12 h.

### Tolerability Studies

At present, there are five studies that have evaluated the tolerability of Cannabis derivatives in dogs and cats.

In the study conducted by Gamble et al. (75), with the exception of an overtime increase from baseline in alkaline phosphatase (ALP) levels, no changes were observed, compared to baseline and placebo, in the biochemical–clinical profile of dogs with OA treated with CBD at a dose of 2 and 8 mg/kg twice a day for 28 days.

Similarly, McGrath et al. (74) found only a significant increase in serum ALP activity in epileptic dogs treated with 2.5 mg/kg every 12 h for 12 weeks.

In a previous study, McGrath et al. (73) assessed the tolerability of CBD as microencapsulated oil beads, CBD-infused oil, or CBD-infused transdermal cream (10 healthy beagles/formulation) at a dose of 10 or 20 mg/kg/day for 6 weeks, finding only elevations in serum ALP in some dogs.

Also, Deabold et al. (72) provided a preliminary assessment of CBD safety and adverse effects during 12-week administration using a hemp-based product in eight healthy dogs and cats after the administration of 2 mg/kg orally twice daily for 12 weeks. Serum chemistry showed no clinically significant alterations; however, one cat showed a persistent rise in alanine aminotransferase (ALT) above the reference range for the duration of the trial. Cats showed adverse effects of excessive licking and head-shaking during oil administration.

Finally, Vaughn et al. (81) have determined the safety, tolerability, and adverse events (AEs) (by clinical observations, physical examinations, complete blood counts, clinical chemistry) of 10 escalating doses of three cannabis oil formulations, containing predominantly CBD, THC, or CBD and THC (1.5:1) vs. sunflower oil placebo and medium-chain triglyceride oil placebo, in twenty healthy beagle dogs (4 subjects/group). The CBD oil was delivered up to ~62 mg/kg, the THC oil up to ~49 mg/kg, and the CBD/THC oil up to ~12 mg/kg CBD + 8 mg/kg THC. Overall, dogs tolerated the dose escalation of the CBD oil well, experiencing only mild AEs (94.9% of all AEs). Moderate AEs (4.4% of all AEs) and severe AEs (0.8% of all AEs) occurred across the two groups receiving oils containing THC (CBD/THC oil or THC oil) and were mainly characterized by lethargy, hypothermia, or ataxia. These data show that CBD-predominant oil formulation appears to be well-tolerated in dogs and safer than oil formulations containing higher concentrations of THC.

As the THC is concerned, the minimum lethal oral THC dose for dogs has been established in more than 3 g/kg (82, 83). The median lethal dose, commonly known as LD50, has not been established in dogs or cats (82).

### Legal Restrictions

With regard to the therapeutic use of Cannabis derivatives (either pure CBD oil or oil extracts of dried flowers of Cannabis cultivars specially grown for this purpose by authorized factories), the laws and regulations are substantially different among countries, with some of them (Canada, some states in the United States and some European countries) approving several cannabinoid-based medicines that the human physicians can prescribe for specific indications (84). As a consequence, by virtue of the EU Directives on the use of the veterinary drugs and in particular of the cascade possibility, in those European countries where Cannabis is allowed for human therapy the veterinary practitioner can prescribe galenic formulations based on Cannabis derivatives, extemporaneously prepared by the pharmacist.

It must be stressed out that although some cannabis products containing CBD have been introduced on the market as animal dietary supplements, to date the EU and USA laws have not approved products labeled to contain “hemp” that may also contain CBD for any use in animals, and the European Medicine Agency and the U.S. Food and Drug Administration cannot ensure the safety or effectiveness of these products.

## Discussion and Conclusions

Hemp is a very useful plant, of which all parts can find a practical use.

Besides the use of external fibers of the stem in the textile, plastic, paper, and construction industry and of seeds in human nutrition, specific components of the plant can find application in animal nutrition as well as in animal therapy.

The aforementioned studies indicate that hempseeds and their derivates may be included in diets of livestock as a good source of crude protein and essential fats, without particular changes in growth performance (85).

With regard to the nutraceutical properties of Cannabis derivatives, several effects have been postulated as a consequence of their addition to basal feed, such as a reduction in the rate of tibia deformation (in broilers and hens), a better serum lipidic profile, a protective effect on the development of liver diseases, an antimicrobial activity, the promotion of an antioxidative systemic status, and an anti-inflammatory activity (13, 14, 20, 22, 34). All these effects need however to be confirmed by further studies.

A not less important feature of the integration of hempseed products in animal feed is the positive effect, from a human health point a view, of fatty acid composition of meat, eggs, and milk, with an enrichment in ω-3 PUFA content (10, 16, 19, 21, 24, 27, 32). Indeed, ω-3 PUFA are considered useful to prevent cardiovascular diseases, cancer, diabetes, obesity, arthritis, and neurological disorders (86, 87). At present, human Western diets are characterized by an excessive intake of ω-6 PUFA and a very high ω-6 to ω-3 ratio (often close to 10:1 and sometimes above 20:1) (88) that can promote the development of many diseases (such as coronary heart disease, hypertension, cancer, inflammatory, and autoimmune diseases) and interfere with normal brain development and cognitive functions. A more balanced ω-6/ω-3 ratio of 3:1 to 4:1 could prevent the pathogenesis of many diseases induced by today's Western diets (86). The use of hempseed products in farm animals can be useful to obtain foods of animal origin with beneficial effects for human health. As an example, the inclusion of hemp seeds in the diet of hens decreased the ω-6/ω-3 ratio in egg yolk from 11:1, observed with the control diet, to 3:1 (21).

To obtain a more comprehensive picture of the real benefits of hemp products in animal nutrition, further studies aimed to improve the knowledge about growth performances, nutraceutical effects, and influence on fatty acid composition of food of animal origin are advocated.

The last part of this review briefly summarizes the composition, localization, and physiologic roles of the endocannabinoid system then sums up the studies published at present on the efficacy, pharmacokinetics, and tolerability with regard to the use of Cannabis derivatives as drugs in dogs and cats. Indeed, in the last years, the interest in the pharmacological actions of *C. sativa* in the contest of several pathologic disorders affecting humans and animals has increased considerably, and several research projects aimed to evaluate in laboratory and companion animals as well as in humans the benefits of this promising plant have been granted. Unfortunately, the legal restrictions of some countries represent a limit to the experimentation in both human and veterinary fields, together with the difficulties in the treatment's standardization (due to the extreme variability in the active principles content in different cultivars) and in the execution of randomized, placebo-controlled, blind clinical trials. Therefore, the small number of works published since now and the limitation of conclusive results restrict the discussion. From the articles published so far, it seems that Cannabis derivatives represent a promising pharmaceutical possibility for the treatment of various pathologies affecting dogs and cats. In particular, CBD, alone or together with the Cannabis phytocomplex, seems to be very useful in managing pain (especially neuropathic, OA, and cancer pain) and epilepsy. Besides the very few scientific articles that demonstrate the efficacy of Cannabis derivatives in such pathologies, there are several veterinary empirical experiences attesting to a fair level of efficacy and safety of Cannabis derivatives when used to manage OA pain or idiopathic epilepsy refractory to conventional treatments in dogs and cats.

With regard to OA patients, all subjects (both dogs and cats) whose clinical history has been anecdotally reported had received conventional NSAIDs or corticosteroids, whether or not associated with gabapentinoids and/or other analgesic adjuvants, without obtaining a valid and lasting benefit or absence of side effects. Depending on pain severity, veterinarians' confidence with the cannabis-based compounds, formulations' availability, and availability and reliability of the owner, galenic formulations containing different ratios of THC and CBD, pure CBD oils, or CBD-based nutritional supplements (5 or 10% CBD—with or without the phytocomplex) were administered *per os* alternatively or in combination. In dogs, CBD doses ranged from 0.05 to 1 mg/kg bid or tid, while in cats 2.5 mg (~0.5 mg/kg) sid were used; in both species, THC doses ranged from 0.05 to 0.25 mg/kg/day (divided into two administrations). In all cases, the principle of “*start low, go low*, and *stay low*” were applied, and in all cases, doses were titrated to effect according to the patient's response. In most of the treated subjects, an improvement in the animal's mood and activity was detected within a few days from the treatment beginning: this improvement was characterized by a greater interest for the environment, greater willingness to play and interact with other animals/humans, increased range of joint motion, less reaction to palpation of the painful joint, and reduced scores obtained through the use of pain scales. In no case was the appearance of side effects highlighted, except for a slight sedation that occurred mainly in those cases in which the initial dose of phytocannabinoids was too high. These results, although limited by the lack of a scientific and rigorous approach, let us speculate on the efficacy and safety of Cannabis for the management of OA pain in dogs and cats (89).

As regard the experiences in dogs suffering from idiopathic epilepsy refractory to conventional pharmacological treatments (generally phenobarbital, potassium bromide, and levetiracetam), there are several clinical evidences of benefits obtained after treatment with Cannabis derivatives. Depending on severity and the frequency of the epileptic seizures, formulations' availability, and owner's availability and reliability, galenic formulations containing a 1:1 THC-to-CBD ratio or food supplements based on CBD oil (2.5 to 5%) were used. Oral doses varied from subject to subject (CBD: from 0.1 to 1.5 mg/kg bid; THC: from 0.09 to 0.46 mg/kg bid) and were associated with other anticonvulsant drugs. In most of the treated subjects, a complete remission of the crises or the reduction from cluster to single crises was obtained. However, in some patients the frequency of the crisis remained unchanged, and in rare cases there has even been a worsening of the crisis frequency. The main side effects were ataxia and sedation (especially with THC based formulations). Despite the not unequivocal results (probably due to the different forms of epilepsy manifested by individual patients, the different formulations used, and the dosages empirically provided), considering that many of the treated subjects responded positively, it is possible to conclude that treatment with Cannabis derivatives would appear to be quite promising (90).

It is in the authors' hope that appropriate clinical trials may be conducted in the near future to confirm the effectiveness of Cannabis in all those pathologies for which it could be pharmacologically useful. Moreover, also the lack of works that investigate the effect of *C. sativa* on animal welfare in intensive animal farming or industrial livestock production should be fulfilled.

## Author Contributions

GR and AD equally contributed to the manuscript preparation. All authors listed have made a substantial, direct and intellectual contribution to the work, and approved it for publication.

## Conflict of Interest

The authors declare that the research was conducted in the absence of any commercial or financial relationships that could be construed as a potential conflict of interest.
